# Integrated PIN modulator and photodetector operating in the mid-infrared range from 5.5 μm to 10 μm

**DOI:** 10.1515/nanoph-2023-0692

**Published:** 2024-01-11

**Authors:** Thi Hao Nhi Nguyen, Victor Turpaud, Natnicha Koompai, Jonathan Peltier, Stefano Calcaterra, Giovanni Isella, Jean-René Coudevylle, Carlos Alonso-Ramos, Laurent Vivien, Jacopo Frigerio, Delphine Marris-Morini

**Affiliations:** Université Paris-Saclay, Centre de Nanosciences et de Nanotechnologies, 91120 Palaiseau, France; L-NESS, Dipartimento di Fisica, Politechnico di Milano, Polo di Como, Via Anzani 42, 22100 Como, Italy

**Keywords:** silicon photonics, electro-optical modulator, photodetector, silicon-germanium, PIN diode, mid-infrared

## Abstract

This study reports the experimental demonstration of the first waveguide-integrated SiGe modulator using a PIN diode operating in a wide spectral range of the mid-infrared region. At the wavelength of 10 µm, an extinction ratio up to 10 dB is obtained in injection regime and 3.2 dB in depletion regime. High speed operation is obtained, up to 1.5 GHz. Furthermore, the device can also operate as an integrated photodetector. Photodetection has thus been characterized from 5.2 µm to 10 µm wavelengths showing an internal responsivity around 1 mA/W, and a 3 dB electro-optical bandwidth of 32 MHz. These results show a significant advancement in integrated photodetectors and electro-optical modulators for mid-infrared spectroscopy.

## 1 Introduction

Silicon (Si)-based integrated photonic circuits operating in the mid-infrared spectral range are highly attractive to develop on-chip mid-infrared spectroscopy systems with many applications in environment monitoring, medical diagnosis or even free-space communications [[Bibr j_nanoph-2023-0692_ref_001] [Bibr j_nanoph-2023-0692_ref_002] [Bibr j_nanoph-2023-0692_ref_003] [Bibr j_nanoph-2023-0692_ref_004]. Furthermore, Si photonics paves the way for monolithic fabrication at the wafer scale, offering cost-effectiveness and higher reliability. The integration of active photonic components such as electro-optical modulator (EOM) and photodetector are essential for sensing systems. Indeed, high-performance EOM is a key device for synchronous detection to enhance the sensitivity of the detection in the mid-infrared range, where molecules exhibit robust absorption features. Furthermore, the development of high-speed RF-driven optical modulator is expected to be of great significance for the development of dual comb spectroscopy, a technique that enables the precise scanning of optical spectrum with high frequency resolution [6], [7]. Interestingly, free-space telecommunications could also take advantage from the development of optical modulator with high-speed characteristics in the mid-IR atmospheric transparency windows (i.e., 3–5 µm and 8–14 µm).

Development towards high-performance silicon photonics based EOM in the mid-IR has advanced rapidly recently, and researches have focused on quantum-confined stark effect (QCSE) or free carrier plasma dispersion (FCPD) effect to achieve optical modulation at the shorter-wave mid-IR spectral range (wavelengths from 2 µm to 4 µm) [8], [9]. In the longer-wave spectrum, Si-based photonics rely on Ge-on-Si, SiGe alloy or SiGe graded on Si substrate, benefiting from low propagation loss due to the broad transparency windows of Ge up to 16 µm [[Bibr j_nanoph-2023-0692_ref_010] [Bibr j_nanoph-2023-0692_ref_011] [Bibr j_nanoph-2023-0692_ref_012] [Bibr j_nanoph-2023-0692_ref_013]. Indeed, the simulated study of QCSE exploiting intersubband transitions in Ge/SiGe quantum well reports intensity modulation at 10 µm wavelength, showing 1 dB extinction ratio and speed reaching few tens of GHz along short 1-µm-long device [15]. Besides, FCPD has been used to demonstrate mid-infrared integrated optical modulators. A Ge-on-Si PIN modulator has been reported first at 3.8 µm and 8 µm wavelengths [16]. Targeting wideband spectral operation, Schottky diodes embedded in SiGe graded waveguides have then been exploited, as the modulation of the depletion width below the Schottky contact allows for modulating the absorption of the optical mode. Modulation has been reported from 6.4 to 10.7 µm wavelength in both depletion and injection schemes [17]. Based on this result, subsequent efforts have resulted in an enhancement of the modulation speed, achieving high-speed operation up to 1 GHz [18]. However, the use of the Schottky contact on top of the waveguide is responsible for a trade-off between propagation loss and modulation depth. In the work reported hereafter, it will be shown that this tradeoff can be overcome by the use of a PIN diode, which allows to push the modulated region far from the top contact. High-efficiency modulation and high speed operation will be shown hereafter.

In parallel, it can be noted that while bulk mid-IR photodetectors are currently largely developed, mainly relying on III–V materials multiquantum well heterostructure (e.g., InGaAs and GaSb) [[Bibr j_nanoph-2023-0692_ref_018] [Bibr j_nanoph-2023-0692_ref_019],narrow band gap materials (HgCdTe) [21], or wideband bolometers, there is only sparce demonstrations of waveguide integrated mid-IR photodetectors. The development of Si-based GeSn photodetector permits direct band gap absorption with high detectivity up to 3.65 µm wavelength [22]. Defects mediated photodetection has been investigated in PIN junctions in SOI waveguides, enabling sub-band gap absorption in the short-wave infrared spectrum (*λ* < 2.5 µm) [23]. The same effect has also been used to achieve photodetection exploiting a PIN junction embedded in a germanium on Si (GOS) waveguide at 3.8 µm wavelength [24] and using a Schottky junction in a SiGe graded waveguide on Si substrate, operating from 5 µm to 8 µm wavelength [25]. Interestingly, it will be shown in the following that the PIN modulator investigated in this study can also be used as an efficient photodetector operating at room temperature in a broad spectral range. Photodetection will be shown from 5.2 to 10 µm wavelength and high speed operation of this device will be demonstrated, with a 3 dB bandwidth of 32 MHz.

Overall, the demonstration of high-performance integrated electro-optical modulator and photodetector operating in the deep mid-IR wavelength range, using PIN diode embedded in SiGe waveguides paves the way for the development of a complete integrated platform for spectroscopic applications.

## 2 Results and discussion

### 2.1 Modulator and photodetector design

**Figure 1:**
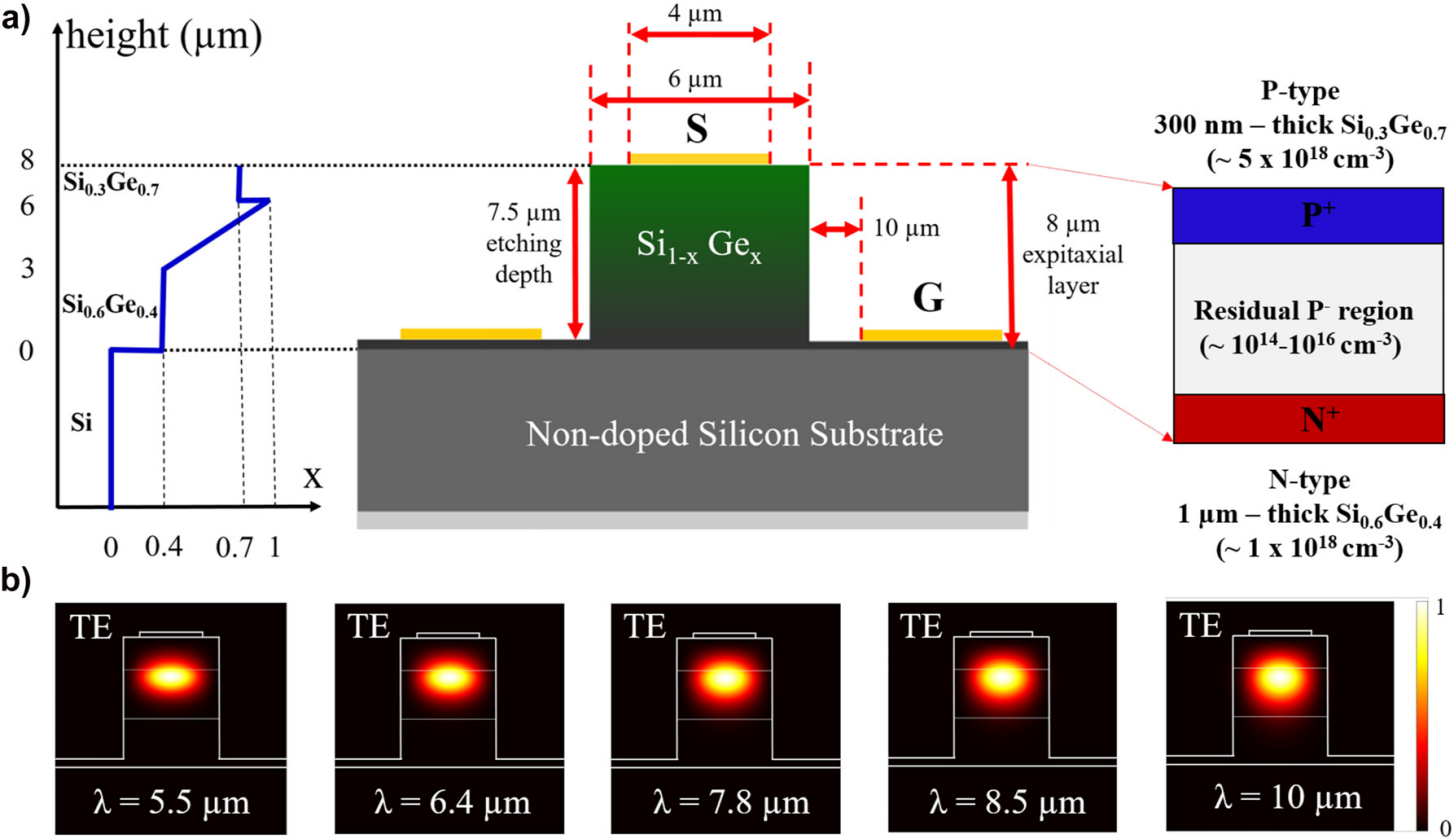
Design of the integrated PIN-based modulator and photodetector. (a) Cross-sectional schematic view of the active region embedded inside the SiGe waveguide on Si substrate. Left: Ge concentration variation in the SiGe alloy along the vertical direction. Right: schematic representation of the PIN junction device illustrating the doping concentration in P an N parts. (b) Optical mode calculations show normalized electric field intensity (TE polarization) in the mid-infrared spectral domain corresponding to the wavelengths of 5.5 µm, 6.4 µm, 7.8 µm, 8.5 µm and 10 µm.

The schematic view of the EOM is depicted is Figure 1a, with the refractive index profile on the left part, and the doping profile on the right part. The design of the electro-optical modulator comes from a tradeoff between optical and electro-optical properties of the device. Indeed, a PIN diode is used to inject/deplete carriers in the core of the waveguide. As the refractive index of silicon germanium (SiGe) alloy is proportional to its germanium (Ge) concentration, the waveguide core can be designed to minimize the overlap of the mode with doped and metallic regions. To this end, a 3-µm-thick layer of Si_0.6_Ge_0.4_ is epitaxially grown on a non-doped Si substrate, followed by a 3-µm-thick graded Si_1−*x*_Ge_*x*_ layer. In this graded layer, *x*, the fraction of Ge in the SiGe alloy, linearly increases from 40 % to 100 %. A 2-µm-thick layer of Si_0.3_Ge_0.7_ is finally grown on top of this structure, to effectively confine the optical mode within the photonic waveguide and away from the upper metallic part. A PIN vertical diode embedded inside the integrated waveguide is used to exploit the free carrier plasma dispersion effect. The first 1-µm-thick Si_0.6_Ge_0.4_ layer from the bottom is thus heavily doped with phosphorus, with a doping concentration of 1 × 10^18^ cm^−3^, forming the N-type region. Conversely, the upper 300-nm-thick layer of Si_0.3_Ge_0.7_ is P-type, doped with boron at a concentration of 5 × 10^18^ cm^−3^. The region sandwiched between the N and P regions is non-intentionally doped. It will be shown later, by the comparison between experimental results and numerical simulations, that this non-intentional doped region possesses a residual P-doped behavior, with doping concentrations ranging between 10^14^ and 10^16^ cm^−3^_._ While the total thickness SiGe layer is 8 µm, an etching depth of SiGe waveguide is 7.5 µm to define the waveguide, so it can be used in the same time to take the bottom contact on the N-doped region. Coplanar waveguide electrodes are used for radio-frequency (RF) operation. The signal line is a 300 nm-thick and 4 µm-wide gold layer on top of the 6 µm-wide waveguide. Ground lines are defined on each side of the waveguide, on the N-doped layer.

Simulated optical modes in TE polarization are reported in Figure 1b at the wavelengths of 5.5 µm, 6.4 µm, 7.8 µm, 8.5 µm and 10 µm. As expected, optical modes are well-confined in the middle part of waveguide and positioned far away from the upper metallic contact.

### 2.2 Fabrication process

**Figure 2:**
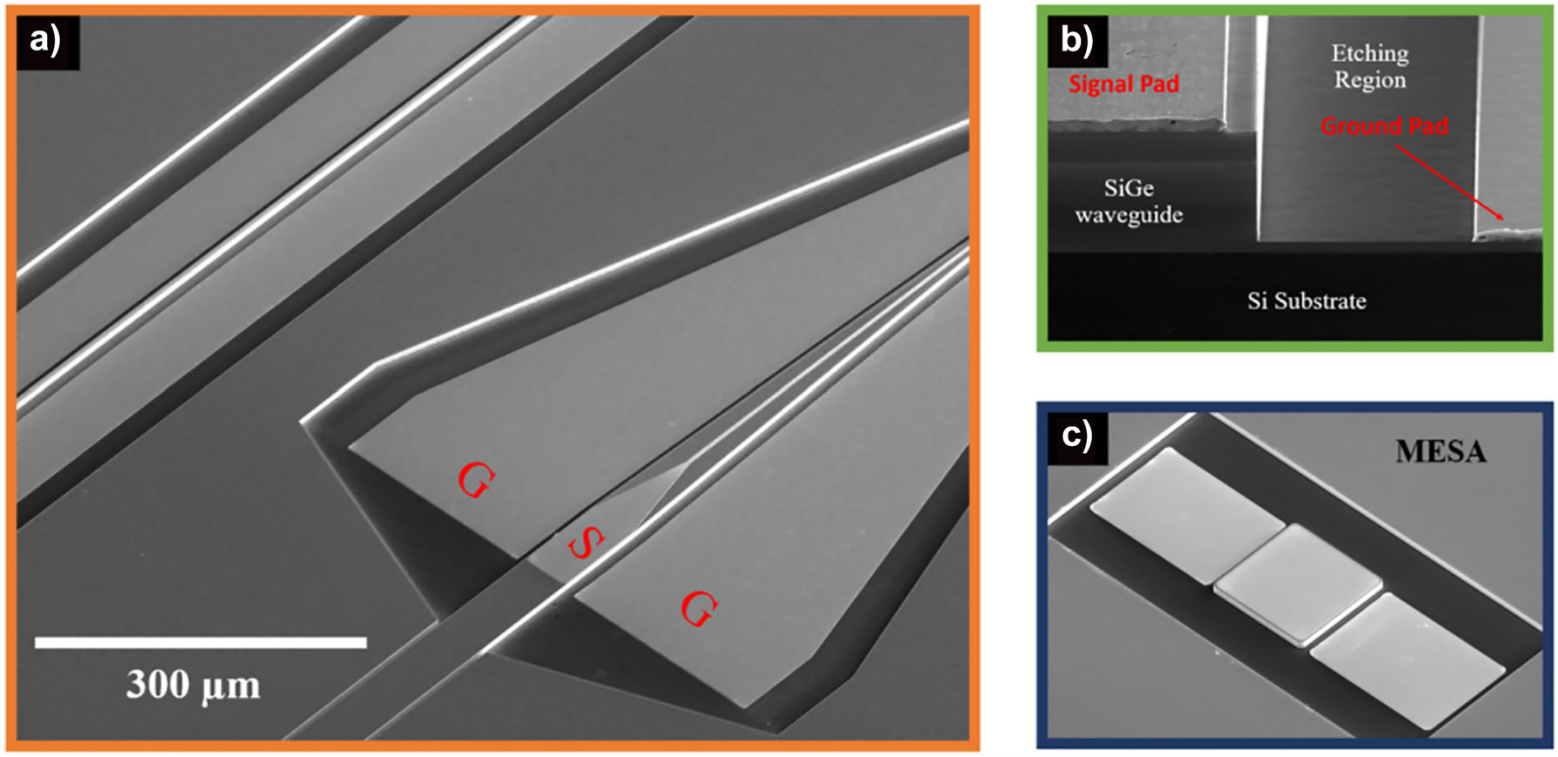
Scanning electron microscope (SEM) images illustrating the fabricated device. (a) Top-view of mid-IR integrated photonic circuit. (b) Lateral view in the active region, showing the presence of signal and ground pads located on top, as well as the etched waveguide region, respectively. (c) Square-shaped MESA device fabricated to characterize the electrical performance of the PIN diode.

The epitaxial growth of the 8-µm-thick SiGe layer on a non-doped Si substrate is performed by low energy plasma enhanced chemical vapour deposition (LEPECVD) [26]. PH_3_ and B_2_H_6_ gases are used to obtain the N and P doped region, respectively. The fabrication process of the PIN-based EOM required 5 lithography steps. E-beam is used as a versatile tool available for research purpose, but there is no critical dimension or alignment that would prevent the use of deep-UV lithography to realize the device. The initial step consists in patterning the metallic strip line on top of the device, followed by the deposition of a metallic layer including 10 nm of titanium (Ti) and 300 nm of gold (Au) via metal evaporation method. In the second lithography step, the photonic waveguide is defined, followed by waveguide etching by inductively coupled plasma reactive ion etching (ICP-RIE), to achieve 7.5 µm etching depth. With such steep deep etching depth, challenges arise in depositing metal for the ground contact in the etched region. Hence, the strategy that has been employed uses 3 successive lithography, and thus 3 layers of 1.4-µm-thick photoresist to ensure the proper coverage of the optical waveguide sidewalls. Finally, the ground contact is created by depositing the same stack layer of Ti/Au. The scanning electron microscope image of the fabricated fully integrated electro-optical modulator is provided in Figure 2a. Notably, Figure 2b illustrates clearly both signal and ground contacting pads located on top of waveguide and in the etched region of the EOM device, respectively.

**Figure 3:**
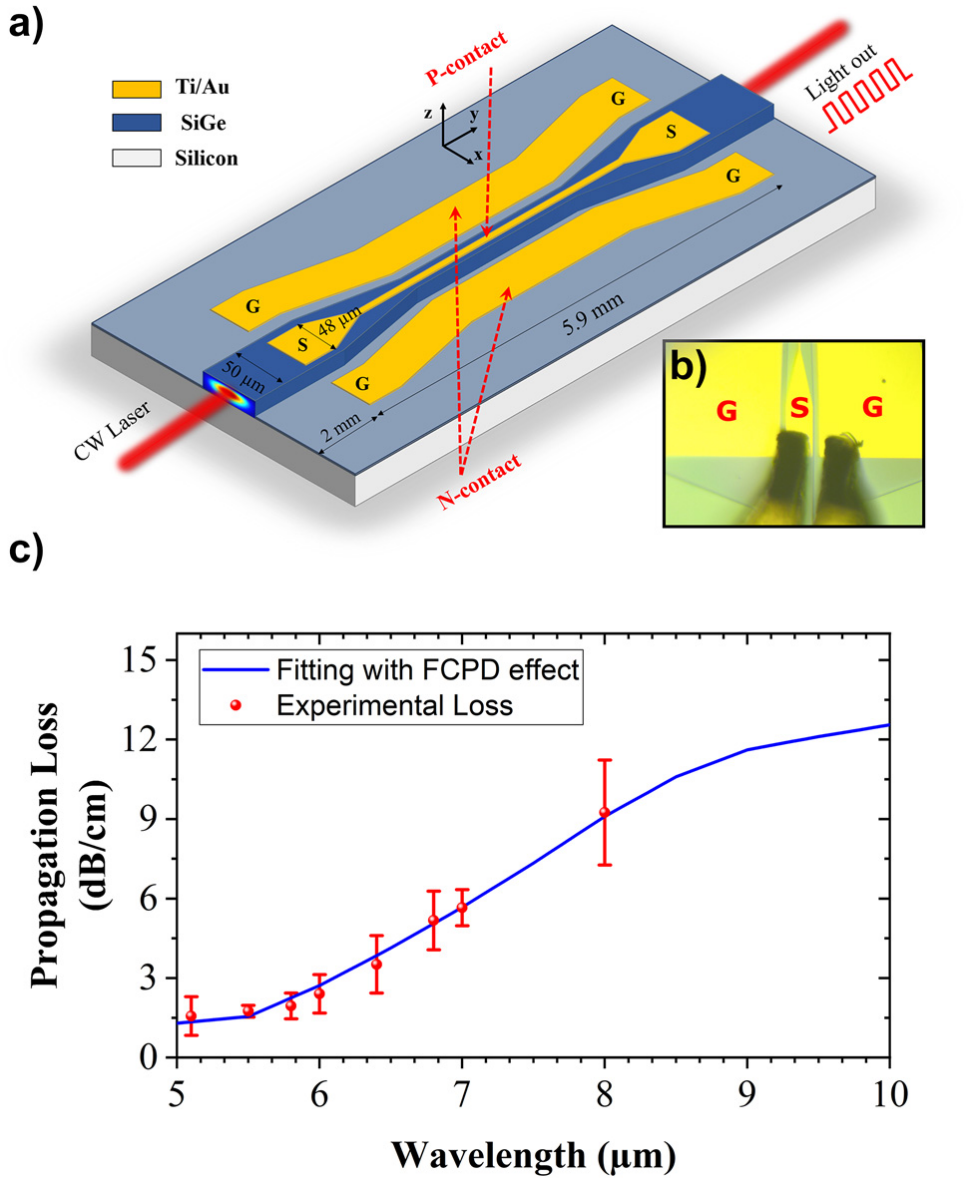
Mid-IR integrated SiGe photonic chip. (a) 3D schematic view of SiGe optoelectronic device which works as both an integrated mid-IR electrical-optical modulator and a photodetector. (b) Microscope image in the electrical contact area where the RF probe has been intentionally tilted to facilitate the access of probes to the signal and ground pads. (c) Experimental propagation loss (TE polarization) in the PIN doped SiGe waveguide (without any top metallic layer) is plotted with fitting data based on FCPD effect (blue solid line) for the wavelengths ranging from 5 µm to 10 µm.

Figure 3a shows the 3D schematic view of the mid-IR waveguide-integrated SiGe based on PIN diode. The input waveguide width is 50 µm to facilitate light coupling. The waveguide width is then reduced down to 6 µm using a 600-µm-long taper. To accommodate the electrical access via the external RF probes, a 48 µm-width signal pad is used, followed by a 100 µm-long taper down to the 4 µm-wide metallic line on top of the waveguide. The gap between the top signal line and the ground line is 12 µm. To ensure electrical contact, Signal Ground (SG) probes are used, intentionally tilted, as shown in Figure 3b. The overall length of the SiGe electro-optical modulator/photodetector is 5.9 mm.

### 2.3 Electrical characterization

**Figure 4:**
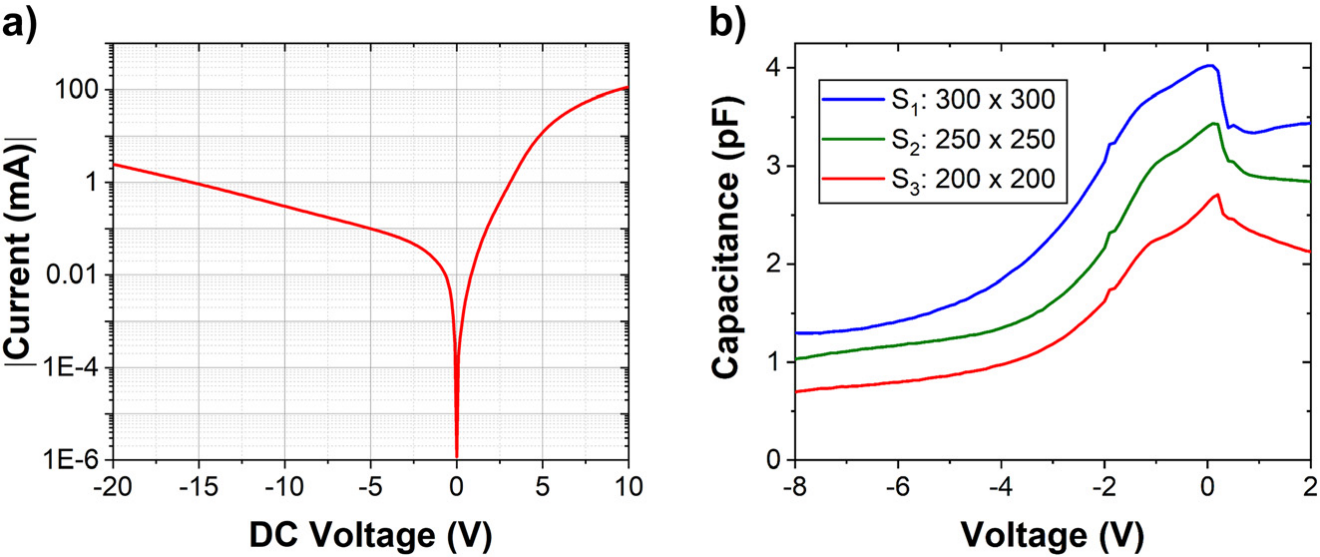
Electrical characterization of the PIN-based SiGe EOM. (a) *I*–*V* characterization in the 5.9 mm long device. (b) Evolution of the capacitance in test structures (squared MESA) with different dimensions, as a function of the applied voltages (length of 200 µm, 250 µm and 300 µm).

Electrical behavior of the PIN diode is evaluated through current/voltage (*I*–*V*) and capacitance/voltage (*C*–*V*) characterizations, as depicted in Figure 4. The *I*–*V* curve recorded from the 5.9 mm long waveguide-integrated device shows the rectifying behavior in the injection regime. The series resistance extracted from injection configuration is determined to be 236 kΩ μm. Furthermore, *C*–*V* measurement is conducted on the square-shaped MESA structure (illustrated in Figure 2c) on the same chip. This analysis aims to characterize the depletion behavior in the PIN junction. Figure 4b demonstrates the variation of capacitance with the bias voltages applied to MESA on different dimensions (i.e., with the widths of 200 µm, 250 µm and 300 µm). This measurement enables to deduce the capacitance per unit of µm, with 0.06 fF/μm at −8 V bias voltage for the 4-µm-wide metallic line. RC bandwidth limitation of the reverse-biased PIN modulator can be deduced from this measurement. The 3 dB bandwidth of electro-optical modulator is thus expected to be around 11 GHz.

### 2.4 Electro-optical modulator

Two measurements are performed to assess the modulation efficiency of the EOM: the static optical response and the high speed characterization.

In the static regime, the performance of the modulator is characterized using an external cavity-based mid-IR quantum cascade laser (QCL) whose wavelength can be tuned from 5.2 µm to 11.2 µm. The light beam is coupled in and out of the photonic circuit using ZnSe lenses. The EOM is connected to a DC source using RF probes which are in contact with the signal and ground pads.

**Figure 5:**
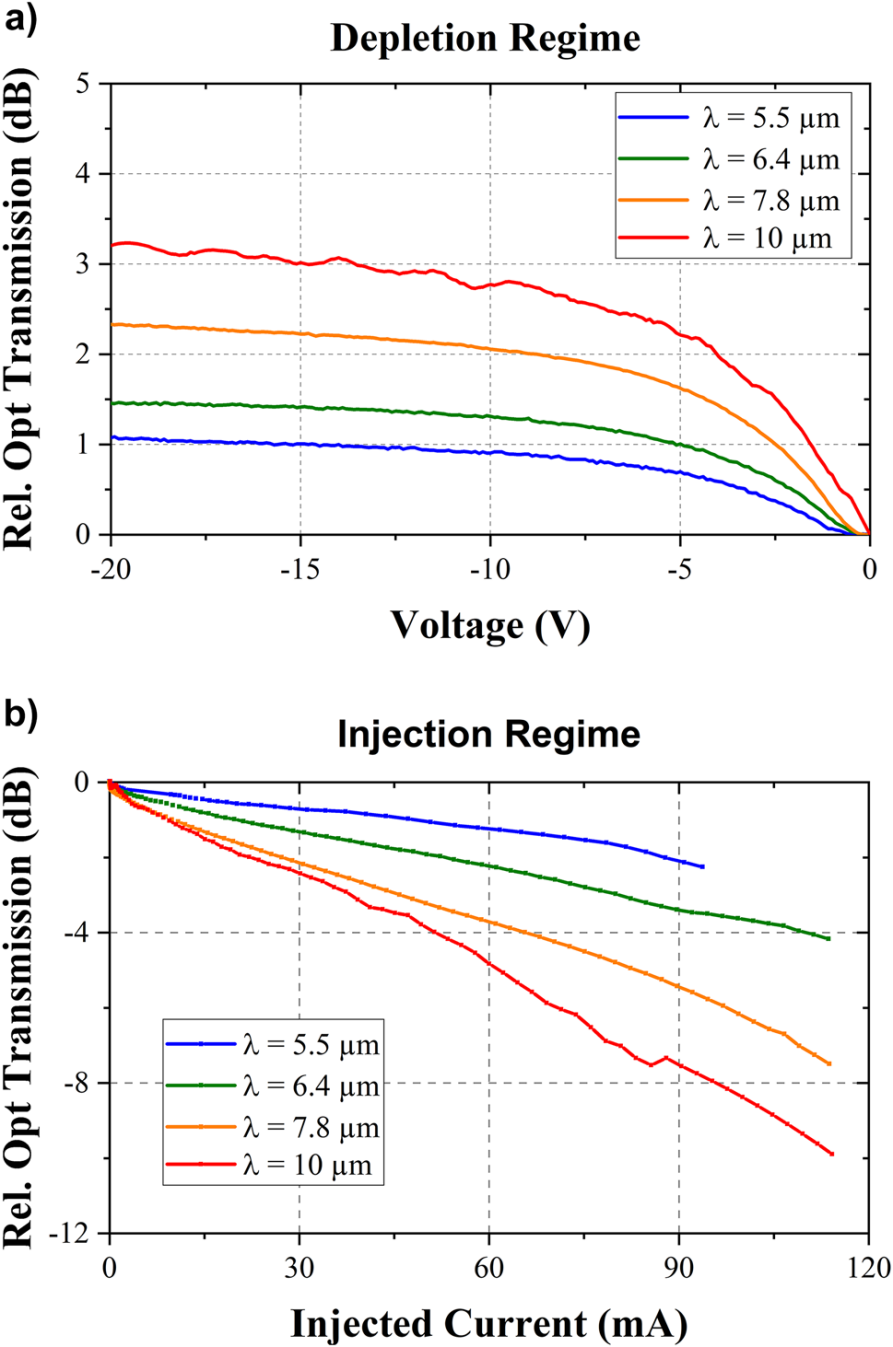
Static characterization of the EOM. (a) In depletion regime (with reverse bias DC voltages). (b) In injection regime (with forward bias DC voltages) at different mid-infrared wavelengths (5.5 µm, 6.4 µm, 7.8 µm and 10 µm).

Figure 5 reports the optical transmission through the 5.9 mm-long modulator in both injection and depletion regimes. The presented data are normalized with respect to the optical transmission at zero-bias voltage. In depletion regime, increasing the bias voltage corresponds to an increase of the optical transmission. Indeed, the space charge region expands within the active region, resulting in a decrease of free carrier absorption. On the other hand, in injection regime, when the current increases, the optical transmission is reduced, as the concentration of free electrons and holes increases in the waveguide, leading to an increase of the free carrier absorption. In both cases, the modulation efficiency increases for longer wavelengths, which is consistent with the theoretical understanding of free carrier absorption in germanium [27]. Interesting, at the wavelength of 10 µm, an extinction ratio of more than 3 dB is obtained in depletion at reverse voltages larger than −15 V, reaching 3.2 dB at −20 V. In injection regime, the extinction ratio reaches 10 dB for a 115 mA current injected in the device.

**Figure 6:**
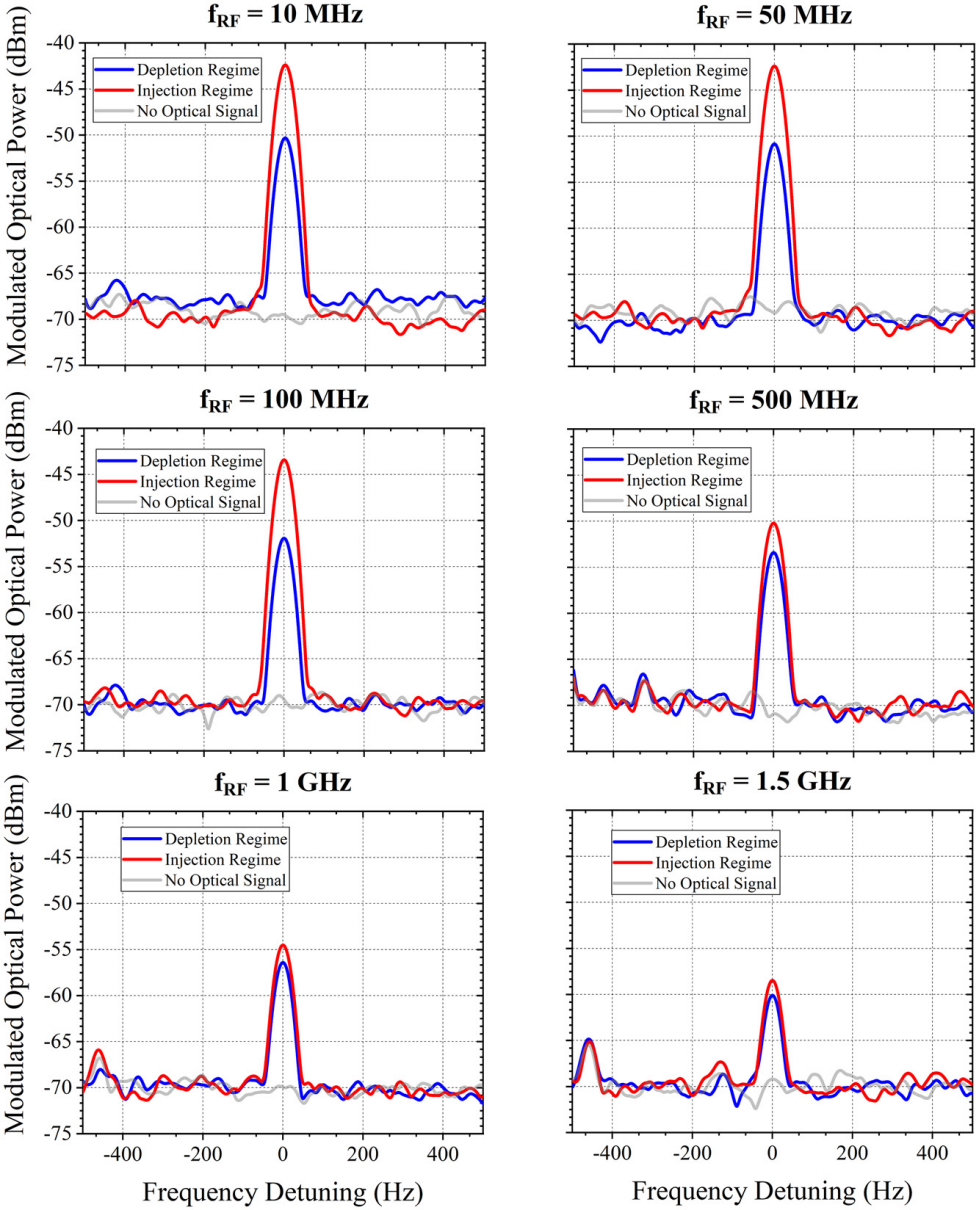
High frequency operation. The peak amplitude of the beat notes measured using the electrical spectrum analyzer at various RF frequencies at the wavelength of 7.8 µm. In depletion regime (blue solid curve), DC bias voltage *V*_DC_ is −4 V and the sinusoid electrical signal has a peak to peak amplitude *V*_PP_ of 8 V. In injection regime (red solid curve), *V*_DC_ is set at 4 V and the *V*_PP_ remains at 8 V. The background noise level (gray solid curve) is the signal measured when the laser is turned-off.

**Figure 7:**
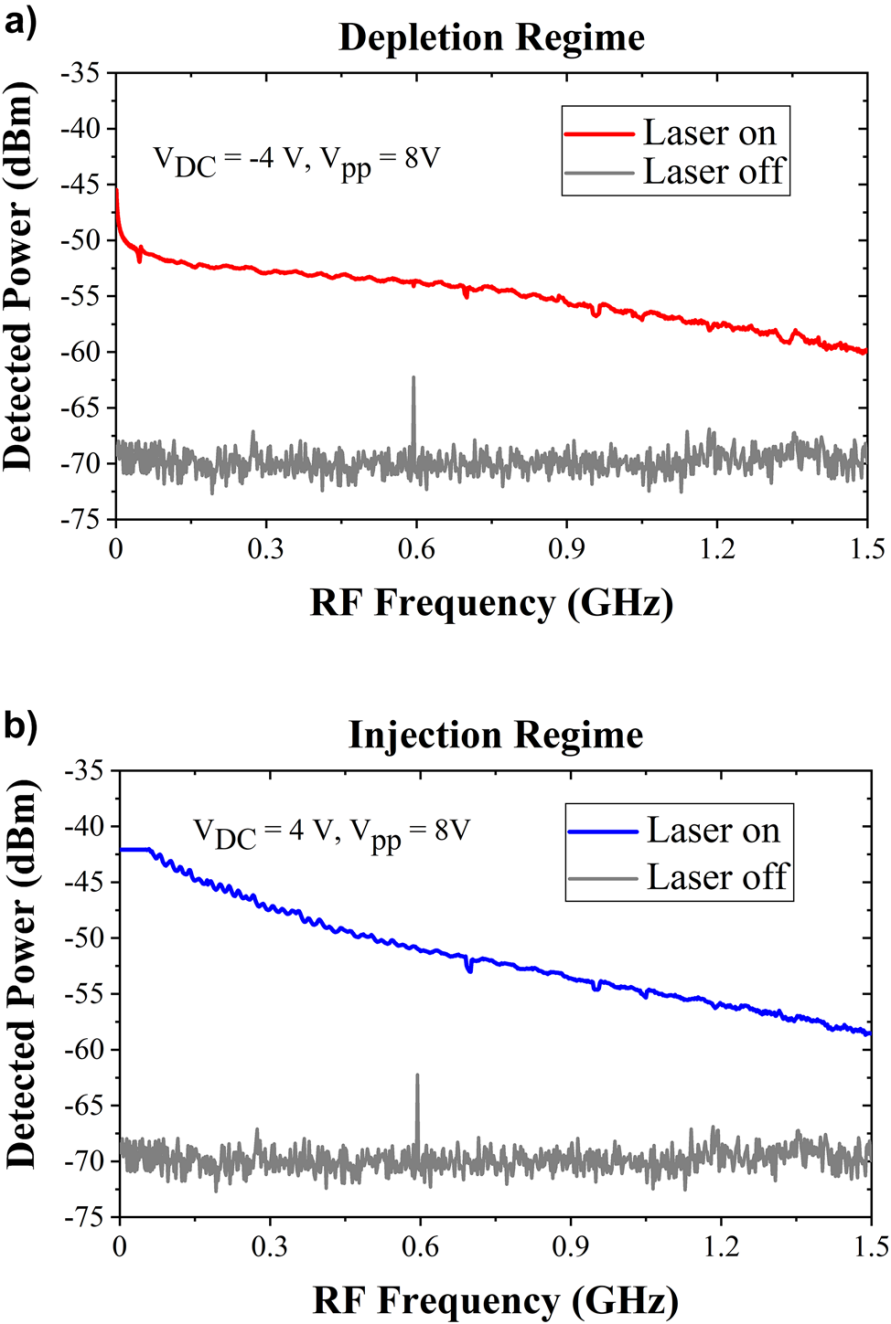
Dynamic characterization of the electro-optical modulator. (a) In depletion regime with *V*_DC_ = −4 V, *V*_PP_ = 8 V and (b) in injection regime with *V*_DC_ = 4 V, *V*_PP_ = 8 V at the wavelength of 7.8 µm.

To evaluate the dynamic operation of the EOM, a continuous-wave mid-infrared quantum cascaded laser is used at the wavelength of 7.8 µm. A 8 V peak-to-peak (8*V*_pp_) RF electrical signal is added to the DC voltage (−4*V*_DC_ in the depletion regime and 4*V*_DC_ in the injection regime) through a bias tee. A second probe is used at the modulator output, connected to a 50 Ω load through a DC block. The modulated optical output is collected and converted to a modulated electrical signal by a commercially available high-speed mid-IR photodetector with a 700 MHz −3 dB bandwidth. A spectrum analyzer is then used to measure the beat note at the modulating frequency. Examples of recorded beat notes at different RF frequencies in both injection and depletion schemes are reported in Figure 6. The figure also illustrates the background noise level, measured when the laser is inactive. Optical modulation can be seen in both injection and depletion up to 1.5 GHz, with a signal-to-noise ratio estimated to be around 10 dB. Figure 7 is reported the evolution of the peak amplitudes of the beat notes as a function of the RF frequencies. In depletion regime, a sharp decrease (4 dB) of the detected signal at low RF frequencies is seen, which could be attributed to the large impedance mismatch originated from the unoptimized electrodes in the device. A rather flat response is then obtained for frequency beyond 100 MHz. In injection regime, the modulated optical power shows a slow decrease as the RF frequency increases, which can be explained by carrier lifetime limitation. The 3 dB bandwidth of the EOM in injection scheme is 200 MHz. However, it is worth noting that in both cases, modulation can be observed up to 1.5 GHz frequency accompanied with low background noise level.

Finally, insertion loss is also an important figure of merits that should be investigated. Two main origins contribute to the modulator loss: (i) the propagation loss of the waveguide itself including the doped region, and (ii) the loss due to the metal on the top of waveguide. Propagation loss is experimentally evaluated using the cut-back method, which involves measuring the transmission of waveguides with different lengths. Figure 3c represents both experimental and simulated propagation loss of the guided mode in the SiGe waveguide including the PIN diode. Besides, loss attributed to the presence of metal is determined via numerical simulation with finite element methods. To the end, the total insertion loss (IL) at zero-bias voltage in the 5.9-mm-long optoelectronic device is evaluated to be at 1.4 dB, 3 dB, 6.2 dB, and 9.3 dB for wavelengths of 5.5 µm, 6.4 µm, 7.8 µm, and 10 µm, respectively.

### 2.5 Photodetector

**Figure 8:**
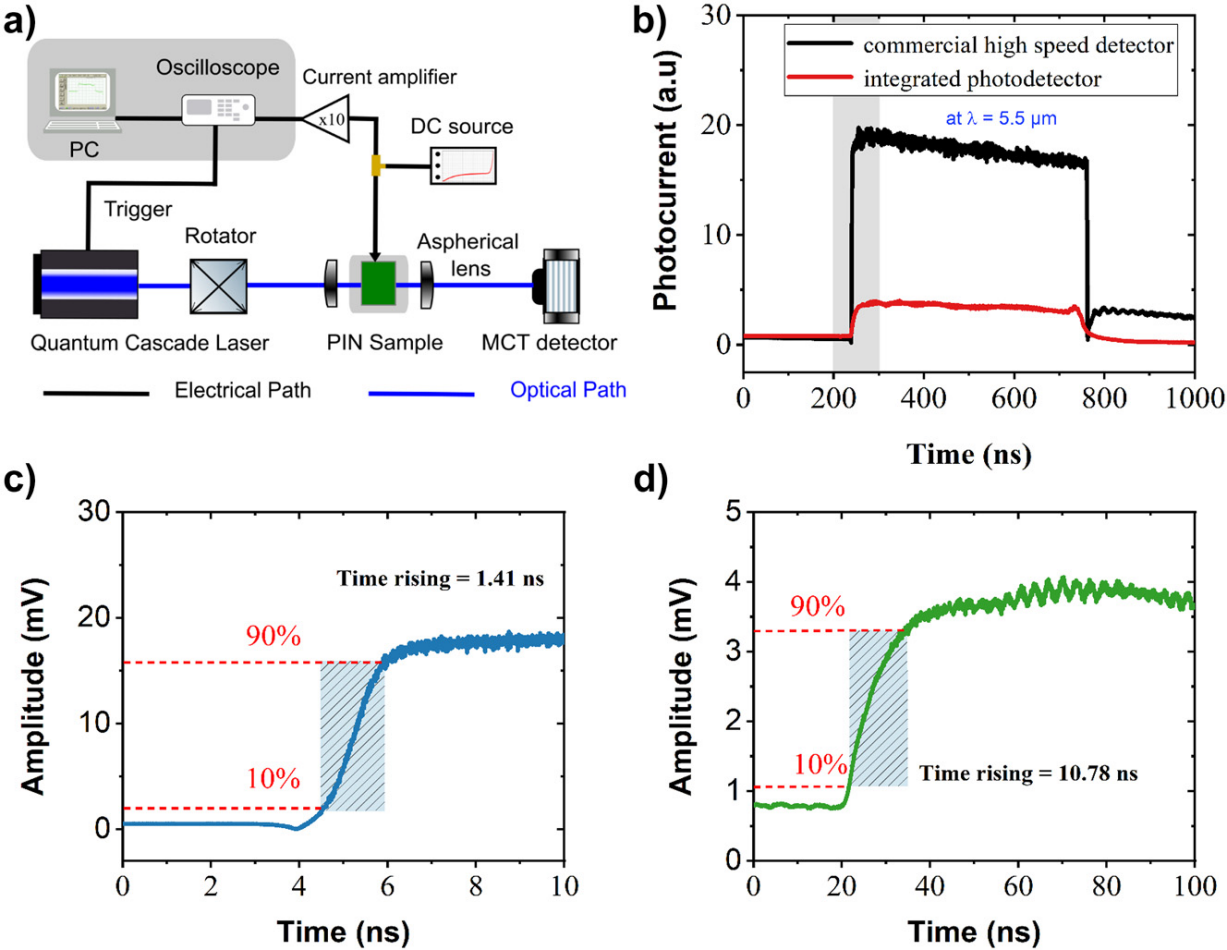
Electro-optical characterization of photodetector. (a) Experimental set-up sketch used to characterize the photocurrent generated in the device. (b) Electrical signal measured in the oscilloscope using a high-speed commercial detector (black), and using the integrated SiGe waveguide (red) at the wavelength of 5.5 µm (amplitude scale is not comparable). (c) Zoom-in the rising time of the signal collected with the high speed commercial detector. (d) Zoom-in the rising time of the signal collected within the integrated SiGe photodetector.

Interestingly, the characterization of the electro-optical modulator revealed that this device can also work as an integrated photodetector in the long-wave spectrum range. Figure 8a shows the experimental set-up used to measure the sensitivity and the speed of this photodetector. A broadband QCL (wavelength from 5.2 to 11.2 µm) is used with a pulsed rate of 100 kHz and a 5 % duty cycle. Photocurrent is measured in the depletion regime. In the following, it is reported for a reversed voltage of −15 V. The photocurrent is first amplified by a current amplifier before reaching the oscilloscope which is synchronized with the pulsed laser. The measurement is done at room temperature, without any temperature control of the photonic circuit. To evaluate the photodetector performances, the amplitude of the photocurrent generated by the integrated SiGe photodetector and its rise time have been evaluated. Furthermore, a comparison has been made using a commercially available high speed detector (bandwidth of 700 MHz), to evaluate the rise time of the optical signal generated by the pulsed laser itself, as shown in Figure 8b. These measurements are performed at the wavelength of 5.5 µm. A zoom-in the rising regions is reported in Figure 8c and d. The rising times of both signals are measured, giving 1.41 ns when using the commercially available photodetector and 10.78 ns using the integrated waveguide SiGe photodetector. It can be deduced that the electro-optical bandwidth of the SiGe photodetector is 32 MHz. Furthermore, from the amplitude of the electrical signal collected, external and internal responsivity can be deduced as followed:

(1) $$R_{\text{ext}}=\frac{I_{\text{pp}}}{P_{\text{ext}\;\text{peak }-\text{ to }-\text{ peak}}}=\frac{I_{\text{pp}}.duty\;\text{cycle}}{P_{\text{ext}\;\text{avearage}}}$$

(2) $$R_{\text{int}}=\frac{I_{\text{pp}}}{P_{\text{int}\;\text{peak }-\text{ to }-\text{ peak}}}$$

while *I*_pp_ stands for the peak-to-peak amplitude of photocurrent, *P*_ext average_ (resp. *P*_ext peak-to-peak_) refers to the measured average (resp. *P*_eak-peak_) external optical power at the entrance of the photonic circuit, out of the chip. *P*_int peak-to-peak_ is the peak-to-peak power coupled in the photonic circuit, evaluated at the entrance of the integrated SiGe photodetector. The calculation of the internal peak power, *P*_int peak-to-peak_, takes into account the coupling loss at the photonic facet (estimated to 7 dB) and the propagation loss along the 2-mm-long photonic waveguide, using measured propagation losses as reported in Figure 3c.

**Figure 9:**
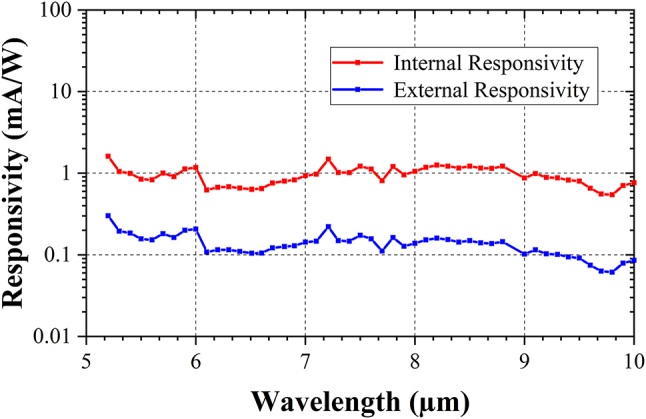
Sensitivity of the integrated photodetector. Internal responsivity (red solid line) and external responsivity (blue solid line) of the SiGe photodetector based on the PIN configuration at a bias voltage −15 V from 5.2 µm to 10 µm wavelength.

Figure 9 shows the evolution of internal and external responsivities of the integrated photodetector as a function of the wavelengths, at the bias voltage of −15 V. Interestingly, the photogenerated current can be observed from 5.2 µm up to 10 µm wavelength, with an internal responsivity, spanning from 0.5 to 1.6 mA/W in this wavelength range. Notably, these responsivities are much higher than the ones achieved using the recently reported Schottky diode SiGe photodetector [25].

## 3 Analysis and discussion

### 3.1 Electro-optical modulator

Numerical calculations are used to model the device, which is an important step towards future performance optimizations. To this end, electrical simulations are used to calculate the distribution of free carriers in the optical waveguide as a function of the applied voltage in the depletion regime. Carrier concentration variation is responsible for variation of the absorption coefficient and refractive index. As there is no interferometric structure in the device, but only straight waveguides, absorption variation only is responsible for optical modulation of the light intensity. This effect is modelled using free-carrier absorption data of pure Ge [26]. Indeed, there is no available data for SiGe alloys, but as a large part of the guided mode is confined in SiGe alloy with the large Ge concentration, it is assumed that this assumption will not affect too much the validity of the results (at 7 µm wavelength, more than 70 % of the optical power is confined in SiGe alloy with Ge fraction more than 70 %). The local distribution of the absorption coefficient in the waveguide as a function of the applied voltage can then be deduced. This resulting map is used to calculate its overlap with the optical mode and to deduce the loss of the optical mode as a function of the applied voltage. As the residual doping of the intrinsic region in the PIN diode was not known, several attempts have been made, considering residual P and N doping. Finally, a very good agreement between the modeling and the experimental results is obtained assuming that the intrinsic region is lightly P-typed, with a carrier concentration of 1.8 × 10^15^ cm^−3^.

**Figure 10:**
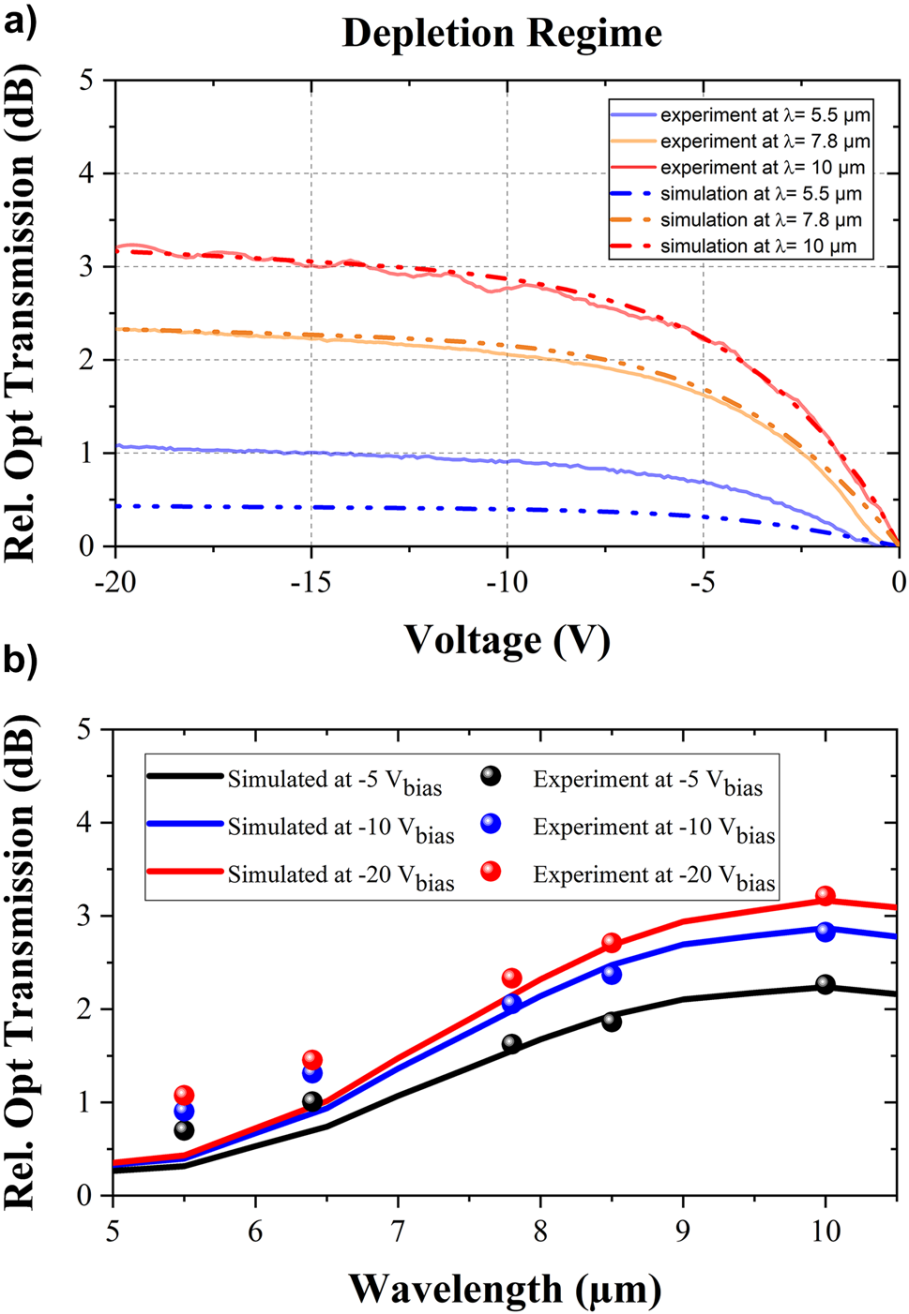
Simulated (dashed line) and experimental (solid line) relative optical transmission are normalized with zero-bias voltage in depletion regime. The intrinsic region is supposed to be lightly P-doped with dopants concentration of 1.8 × 10^15^ cm^−3^. (a) Optical transmission as a function of the bias voltages, for three different wavelengths (5.5 µm, 7.8 µm and 10 µm). (b) Comparison between the simulated extinction ratio (solid line) and the experimental data (dotted mark) as a function of the wavelengths, for three bias voltages (−5 V, −10 V and −20 V).

The comparison between experiments and modeling can be seen in Figure 10. The relative transmission is reported in Figure 10a as a function of the DC reverse bias, at different wavelengths (5.5 µm, 7.8 µm, and 10 µm). A strong agreement is obtained, except at the shortest wavelength of 5.5 µm, which is attributed to the presence of high-order modes. In addition, Figure 10b also reports the simulated and experimental extinction ratio as a function of the wavelengths, at various reverse bias voltages applied to EOM (−5 V, −10 V and −20 V). The general behavior shows a good agreement in the whole wavelength range.

When compared to current state of the art, the performance of the reported mid-IR EOM clearly surpasses previous demonstrations in terms of extinction ratio obtained in a wide spectral range. Further improvement should be considered in terms of device speed, particularly in optimizing the impedance matching of the electrodes along the device.

### 3.2 Photodetector

The results obtained from the integrated PIN SiGe photodetector requires further understanding of the mechanism behind the generation of photocurrent. Indeed, the operating wavelengths are far beyond the bandgap of SiGe alloys, so that even multi photon absorption cannot explain the generated photocurrent. Currently, the main hypothesis relies on sub-bandgap absorption effect, as for the Schottky based SiGe photodetector [25]. Indeed, the epitaxial growth of Ge on Si substrate unavoidably generates defects because of the lattice mismatch between Si and Ge, which is around 4 %. The PIN photodetector is made of a stack of different layers with different composition of SiGe alloys (i.e., Si_0.6_Ge_0.4_, SiGe graded with Ge fraction in SiGe alloy from 40 % to 100 % and Si_0.3_Ge_0.7_), leading to nucleation of defects resulting from misfit dislocations within the active regions. In the depletion regime, the enlargement of the space charge region enhances the overlap of the guided mode with the depleted region, where photocarriers are generated, enabling a more efficient photoresponse.

Further improvements will be dedicated to the optimization of (i) the device responsivity by a better control of the defect concentration within the waveguide and (ii) the speed of the device, starting from a detailed analysis of the limiting mechanism.

## 4 Conclusions

In conclusion, a novel kind of mid-IR integrated electrical-optical modulator has been successful developed by exploiting free carrier plasma dispersion effect in a PIN diode embedded in graded SiGe waveguides. Experimental results have been reported, showing that the EOM operates in broad spectral range from 5.5 µm to 10 µm wavelength. Interestingly, extinction ratio as high as 10 dB in injection regime and 3.2 dB in depletion regime have been obtained at the wavelength of 10 µm. In addition, high speed modulation is demonstrated, using RF signal frequency up to 1.5 GHz. Interestingly, this waveguide-integrated device can also be used as a mid-IR integrated photodetector from 5.2 µm to 10 µm wavelength. The detector exhibits an internal responsivity up to 1.6 mA/W and a 3 dB bandwidth of 32 MHz. These preliminary outcomes are a promising step for future fully integrated mid-IR sensing or free space communication systems.

## 5 Methods

### 5.1 Static and dynamic EOM and PD setup

Static modulation characterization: Static measurement is performed by using a mid-IR quantum-cascaded laser (MIRCAT, Daylight solution) which wavelength can be tuned from 5.2 µm to 11.2 µm. The beam light is coupled in and out of the photonics waveguide by a pairs of ZnSe aspherical lens. This is carried out in the free space configuration, and coupling loss is estimated to be 7 dB/facet. The collimated beam light at the output is collected by MCT detector (DSS-MCT-020, Horiba) which is connected to a lock-in equipment to amplify the optical signal. Modulation is achieved by applying a DC voltage via RF probe which is connected to DC source – meter machine (Keithley 2401).

Dynamic modulation characterization: A continuous-wave mid-IR quantum cascade laser (MIRCAT, Daylight solution) is used to characterize the speed of the electrical-optical modulator. The used wavelength is 7.8 µm, as it corresponds to the wavelength at which the maximum power is coming out of the laser. The output light is collected by an MCT detector (UHSM-I-10.6), Vigo System which is connected to a signal analyzer equipment (MS2830A, Anritsu). DC and AC signals are applied to RF probes through a bias tee with a source-meter equipment (Keithley 2400) and signal generator (MG3694C, Anritsu), respectively.

Dynamic characterization of photodetector: Responsivity and high speed measurement of photocurrent are carried out with a mid-IR quantum-cascaded laser (MIRCAT, Daylight solution) (wavelength from 5.2 µm to 11.2 µm, duty cycle 5 % and repetition rate 100 kHz). The optical beam is butt-coupled to the photodetector. The generated photocurrent is collected by the RF probe, then sent a to current amplifier (DHPCA-100, Femto) before being detected on the oscilloscope (Agilent 86100C DCA-J).

### 5.2 Simulation

Optical profiles are calculated using MODE solver in Lumerical software. Electrical concentration as a function of bias voltage is obtained using CHARGE solver, Lumerical. The overlap between optical profile and charge concentration is deduced by applying Soref’s formula about free carrier absorption with Python code.

### 5.3 Propagation loss experiments and fitting method

To estimate the propagation loss, a set of spiral waveguides are fabricated on a separated sample coming from the same wafer. Propagation loss is measured experimentally using the cut-back method which relies on the measurement of the optical transmission of waveguides of different lengths. This method has been successfully used from 5 to 8 µm wavelength. However, higher losses prevent the use of this method beyond 8 µm wavelength. Among different possible loss contributions, and by comparing the results with the ones of previously fabricated devices without any PIN structure, it was possible to estimate that, in the device reported in this paper, free carrier absorption is dominant compared to other contributions. Therefore, to estimate losses beyond 8 µm wavelength, experimentally measured propagation loss (from 5 µm to 8 µm wavelength) has been fitted using a model based on free carrier absorption. This fitting is then used to estimate propagation loss for wavelengths from 8 µm to 10 µm as reported in Figure 3c.
